# Are viruses important in the plankton of highly turbid glacier-fed lakes?

**DOI:** 10.1038/srep24608

**Published:** 2016-04-20

**Authors:** Fabian Drewes, Hannes Peter, Ruben Sommaruga

**Affiliations:** 1 University of Innsbruck, Institute of Ecology, Lake and Glacier Research Group, Technikerstr. 25, 6020 Innsbruck, Austria

## Abstract

Viruses are ubiquitous in aquatic ecosystems where they significantly contribute to microbial mortality. In glacier-fed turbid lakes, however, viruses not only encounter low host abundances, but also a high number of suspended mineral particles introduced by glacier meltwaters. We hypothesized that these particles potentially lead to unspecific adsorption and removal of free virus from the plankton, and thus significantly reduce their abundance in this type of lake. We followed the distribution of free virus-like particles (VLP) during the ice-free season across a turbidity gradient in four alpine lakes including one adjacent clear system where hydrological connectivity to the receding glacier is already lost. In the glacier-fed turbid lakes, VLP abundance increased with distance to the glacier, but the highest numbers were observed in the clear lake by the end of August, coinciding with the maximum in prokaryotic abundance. Our results suggest that viral loss by attachment to particles is less important than expected. Nevertheless, the relatively lower variability in VLP abundance and the lower virus-to-prokaryote ratio found in the turbid lakes than in the clear one point to a rather low temporal turnover and thus, to a reduced impact on microbial communities.

Viruses are the most abundant biological entities on Earth and occur in most habitats worldwide^1^. Together with phagotrophic protists, viruses are one of the main sources of bacterial mortality in aquatic ecosystems and consequently, they are important drivers of the cycling of carbon and other nutrients^2^. Viral abundance in freshwater lakes usually ranges between 10^5^ to 10^8^ virus-like particles (VLP) mL^−1 ^ (Ref. 3). However, lower VLP abundances are observed in oligotrophic alpine (i.e., located above treeline) lakes. For example, in Gossenköllesee, a lake in the Austrian Alps, VLP abundance ranges between 10^4^ and 10^7^ VLP mL^−1 ^ (Ref. 4,5). Alpine lakes in temperate regions are generally harsh habitats for the biota because incident UV radiation levels and penetration depth are high, mean water temperature is low, and the productive phase of the lake is shortened due to long periods of ice and snow cover^6^.

The rapid ongoing retreat of mountain glaciers and other ice masses is one of the most notable effects of climate change worldwide. The increase in global mean air temperatures leads to enhanced glacial ablation, retreat and volume loss^7^. Glaciers worldwide are affected by global warming and many small ones will potentially disappear within the next decades. For example, in the Alps, glaciers located below 3000 m above sea level are those where area loss is mostly significant^8^. Glacier melting leads to an increase in freshwater discharge and thus, to an enlargement of existing lakes, as well as to an interference with their interannual and seasonal variability in key factors such as water temperature, mineral turbidity, and light penetration^9^. Another important consequence of glacier melting is the creation of many new lakes in the glacier forefield, where topology suitability is met^10^. These newly formed lakes are of great interest for understanding early stages of colonization, community succession, and changes in biodiversity when they shift from turbid to clear states upon hydrological disconnection with the glacier^9^,^11^,^12^. In fact, most lakes in Eurasia and North America originated during the last glacial period ca.10–14,000 years ago^13^ and they likely had similar characteristics to those emerging in glacier forefields nowadays^9^.

Glacier-fed lakes are characterized by a high content of mineral suspended particles called ‘glacial flour’, which result from the erosion at the glacier bedrock and are transported by proglacial streams or runoff^14^,^15^. Glacial flour consists of silt and clay particles ranging between ≤0.002 and 0.063 mm^15^,^16^. Little is known about the effects of glacial flour on lake communities, but a high concentration of those particles can negatively affect keystone planktonic species such as filter-feeding invertebrates^17^ and heterotrophic nanoflagellates^16^. Information on the effect of glacial flour on viruses is not available, but the presence of suspended particles could result in a reduction of viral inactivation rates. For example, phages seem to be more stable when adsorbed onto the surfaces of clays^18^. Further, suspended particles may protect viruses from inactivation by solar ultraviolet (UV) radiation^19^,^20^, and thus may enhance their persistence in the water column. On the other hand, unspecific adsorption to particles and sinking is a major loss factor for viruses^19^,^21^.

Another important factor to consider in turbid glacier-fed lakes is that prokaryotic abundance is low^12^, so that the ratio of suspended mineral particles to prokaryotes is very high^16^. This can eventually affect virus-host encounter rates and thus, viral production^21^. In fact, usually VLP abundance is positively correlated with bacterial and algal abundance, because infection rates increase with host availability^3^,^22^,^23^. Based on these arguments, we hypothesized that the abundance of free VLP will be significantly lower in turbid glacier-fed lakes than in clear ones.

In this study, we assess the occurrence and distribution of VLP during the ice-free season in four lakes representing a turbidity gradient and discuss the abiotic and biotic parameters that may regulate their abundance. The four lakes known as Faselfad (hereafter abbreviated as FAS, Fig. 1) have formed due to the retreat of a mountain glacier^11^,^12^,^16^. Three of the lakes (FAS 1, 3, and 6) are hydrologically connected to the rapidly receding glacier and are turbid (Fig. 1). The fourth lake (FAS 4) has lost its hydrological connectivity with the glacier and is now clear. The lakes are situated along an altitudinal gradient, which also represents the sequence of lake formation, FAS1 being the youngest system.

## Results

### Counting efficiency and influence of free DNA on VLP enumeration

To assess VLP counting efficiency by epifluorescence microscopy, we made an experiment with a known abundance of the T4 phage and with two different concentrations of suspended glacial flour corresponding to 7 and 14 nephelometric turbidity units (NTU). No significant differences in T4 abundance were found between the control and the treatments with different turbidity (ANOVA F_2,6_ = 0.487; p = 0.637) (Supplementary Fig. S1), indicating that viral counts were not underestimated within the turbidity range tested. However, the counting effort was larger with increasing particle abundance. In addition, we tested whether free DNA could interfere with the estimation of VLP abundance. The treatment of lake water samples with DNase decreased VLP counts on average by 12.2% compared to the control (Supplementary Fig. S2), with the highest reduction found in the clear lake FAS 4 (20.2%). Therefore, free DNA was not an important factor at least in the turbid lakes.

### Temporal and spatial VLP distribution

Significant changes in VLP abundance were observed among the lakes over the ice-free season (ANOVA F_3,120_ = 30.8; p < 0.01, Fig. 2). The highest VLP abundance was observed in the clear lake FAS 4 at 14 m depth, on August 1 (1.25 × 10^7 ^VLP mL^−1^), whereas the lowest abundance was observed in FAS 1 at 4 m depth on July 17 (4.38 × 10^5 ^VLP mL^−1^). Considering all four lakes, the lowest VLP abundance was recorded at the beginning of the season soon after the ice cover disappeared (Fig. 2). Later in the season, the average VLP abundance increased in all lakes reaching a maximum in late August. Towards the end of the ice-free season in October, mean VLP abundance decreased again. However, this reduction was mainly observable in the clear lake FAS 4, while VLP abundance remained rather constant in the turbid lakes (Fig. 2). Throughout the season, FAS 1 had on average the lowest VLP abundance compared to the other lakes. Within each lake, VLP abundance was significantly different over the season (FAS 1: ANOVA F_3,16_ = 7.64, p < 0.01; FAS 3: ANOVA F_3,36_ = 114.4, p < 0.01; FAS 4 unequal variance Welch F test F_16,14_ = 40.56, p < 0.01, and FAS 6 ANOVA F_3,24_ = 16.95, p < 0.01).

On the first two sampling dates, variability in VLP abundance in the water column was high in all lakes, whereas depth-related variation decreased towards the end of the season (Supplementary Fig. S3A). On August 1, there was a high variability in the water column of all lakes with a trend towards higher VLP abundance in deeper water layers in the turbid lakes (Supplementary Fig. S4). Variability in VLP abundance in the water column was significantly correlated with that of temperature (R = 0.57, p = 0.02, Supplementary Fig. S3B). In the turbid lakes, we found a significant positive correlation between VLP abundance and water depth in three sampling occasions (FAS 3 on August 1, R = 0.72, p < 0.05; FAS 3 on October 2, R = 0.97, p < 0.01, and FAS 6 on August 28, R = 0.83, p < 0.05). In FAS 4, VLP increased with depth during mid-summer (August 1, R = 0.98, p < 0.01; August 28, R = 0.73, p < 0.05), while no significant correlation (p > 0.05) was found on July 17 and October 2.

### Turbidity and VLP abundance

Throughout the season, FAS 1 was the most turbid lake (ranging between 6.2 and 49.2 NTU), followed by FAS 3 (3.4 to 11.2 NTU), and FAS 6 (1.3 to 6.3 NTU) (Supplementary Table S1). The highest turbidity was measured by the end of August for all three turbid lakes, whereas the lowest values for FAS 3 and 6 were measured in early August, and in mid-July for FAS 1. Lake FAS 4 had very low turbidity over the entire season. There was a significant, but weak negative correlation between turbidity and VLP abundance across the turbidity gradient (correlation for all lakes: R = −0.22, p = 0.02, Fig. 3). However, when the data from FAS 4 were excluded, no significant correlation was found (R = −0.20, p = 0.08). Even under extremely turbid conditions on August 28 (range: 44.2 to 58.7 NTU within the water column), VLP abundances in FAS 1 reached similar levels than under considerably lower particle loads (e.g., 13.6 and 23.3 NTU).

### The coupling between prokaryotic and VLP abundance

Considering all samples, we found significant positive correlations between prokaryotic and VLP abundances for FAS 1 (R = 0.53, p = 0.02), FAS 3 (R = 0.35, p = 0.03), and FAS 4 (R = 0.48, p < 0.01), but not for FAS 6 (R = 0.15, p = 0.46). However, covariation followed a different pattern in the clear and turbid lakes. VLP abundance in FAS 4 followed that of prokaryotes (Fig. 4), with both increasing from mid-July until the end of August and then declining in October. By contrast, in the turbid lakes, VLP abundance increased until beginning of August, though with different magnitude (lowest increase factor found for the most turbid lake FAS 1), and remained at similar levels until the end of the season, whereas abundance of prokaryotes reached the highest values in August and declined afterwards (Fig. 4 and Supplementary Table S1). Chlorophyll-a concentration, a proxy for phytoplankton biomass, increased throughout the season (Supplementary Table S1).

The virus-to-prokaryote ratio (VPR) was significantly higher in the clear lake FAS 4 than in the turbid lakes (Fig. 5, ANOVA, Tukey’s HSD: adjusted p < 0.01).

### Prophage induction

Incubation of composite samples from FAS 3 and FAS 4 with mitomycin C to induce prophages resulted in a moderate induction (average for all data: 10.1 ± 3.5%, Supplementary Fig. S5). Induction rates were higher in August (12.9–13.2%) than in October (6.9–7.1%).

### Relationship of VLP abundance with environmental factors

We assessed the effects of turbidity, DOC, prokaryote abundance, and water temperature on VLP abundance, using multiple regression analysis including data from all depths and sampling dates. Prokaryote abundance, DOC and water temperature were significantly correlated with VLP abundance, whereas turbidity showed no significant effect (Supplementary Table S2).

## Discussion

We expected to find low abundances of free VLP in the glacier-fed lakes due to the presence of high concentrations of suspended particles and the potential removal of viruses by unspecific adsorption^19^,^21^, as well as due to the reduced probability to encounter a host^16^,^21^. Though this was true for the most turbid system FAS 1, our results however, showed that this may not be generally the case for all glacier-fed turbid lakes. In fact, VLP abundance in the turbid lakes FAS 3 and FAS 6 reached similar values as in the clear lake (Fig. 2). Further, VLP abundances in both the clear and turbid lakes were similar to those of a variety of other aquatic systems^3^, such as Lake Superior^24^ (range: 0.2 to 0.9 × 10^6 ^VLP mL^−1^) and lakes in Antarctica^25^ (range: 1.0 to 3.3 × 10^6 ^VLP mL^−1^). Maximum viral abundance (FAS 3: 9.2 × 10^6^ ; FAS 4: 1.25 × 10^7 ^VLP mL^−1^) was also similar to that found in Gossenköllesee, a clear alpine lake^4^ (1.0 × 10^7 ^VLP mL^−1^).

The lack of evidence for a negative effect of mineral turbidity (Fig. 3) on VLP abundance contrasts with reports for coastal marine waters, where a large percentage of the free viruses are removed by attachment to suspended particles^19^,^21^. One important difference, however, is that organic coating of particles and microaggregates are common in coastal marine waters and in more productive lakes, whereas particles of glacial origin have almost no organic coating^16^. This difference likely reduces the probability that viruses irreversibly attach to glacial particles. Moreover, negatively charged particles may weaken or prevent virus adsorption. Glacial particles in the Faselfad lakes are mainly composed of muscovite and illite^16^, which bear a negative charge^26^,^27^. Since phages seem to be negatively charged^28^, viruses in glacial turbid lakes may not efficiently adsorb to particles. Nevertheless, the high numbers of mineral particles and the low host abundance, might still pose a rather unique challenge to accomplish the viral lytic cycle. One alternative mechanism could be that viruses enter a lysogenic life cycle, allowing viral populations to endure such challenging periods. Viruses seem to favor the lysogenic over the lytic cycle when ecosystem productivity is low^22^,^29^, but see Knowles *et al.*^30^. However, despite the oligotrophic condition of the study lakes, we found a moderate induction of prophages (Supplementary Fig. S5), suggesting that the lytic cycle was prevalent. Another interpretation, however, is that we might have not seen higher induction because 24 h incubations with mitomycin C could be too short in this type of lake.

One intriguing difference between the clear and the turbid lakes was that temporal variability was less conspicuous in the latter case (Fig. 2). Particularly, VLP abundance remained relatively constant in the turbid lakes towards the end of the ice-free season, whereas prokaryotic abundance markedly decreased. The more pronounced seasonality of VLP abundance observed in the clear lake FAS 4 than in the turbid lakes may result from a combination of different factors. One is that bacterivory by heterotrophic nanoflagellates in the clear lake may indirectly affect the dynamics of viruses by removing potential hosts, whereas this key planktonic group is absent or present at very low densities in the glacier-fed turbid lakes^16^. Another factor is that free viruses will presumably be more stable and endure for longer periods in the water column of turbid lakes because infectivity decay, for example, by solar UV radiation^22^,^31^ is expected to be less important than in a clear system.

Regarding the spatiotemporal distribution patterns of VLP, we found key environmental factors that covaried over the course of the ice-free season (Supplementary Table S2). For example, prokaryotic abundance, DOC concentration and water temperature were significant factors explaining those patterns (Supplementary Table S2). The abundance of prokaryotes is usually positively correlated with VLP abundance in freshwater and marine systems^22^,^23^,^32^,^33^, however, also weak or no correlations have been reported for freshwater lakes^23^,^31^. Water temperature is known to be a major driver of viral abundance in marine ecosystems^22^,^34^,^35^. Strikingly, the degree of variability in VLP abundance and in temperature across the water column was correlated (Supplementary Fig. S3B), suggesting that thermal structure may be relevant for maintaining relatively constant VLP abundance over time in the turbid lakes. DOC concentration was positively correlated with VLP abundance, which agrees with a study done in Arctic lakes^25^. However, the role of DOC for viral assemblages remains controversial as no clear relationships have been found in lowland and humic lakes^23^,^36^.

The virus-to-prokaryote ratio (VPR) in the FAS lakes (Fig. 5) was within the range reported for other freshwater lakes^3^,^22^,^23^, includig alpine ones^4^,^5^. Yet, in the clear FAS 4 lake, this ratio was actually in the upper range (average ± standard deviation: 53.1 ± 30.2), and occassionally, for example on August 1, it reached >100. The interpretation of this ratio is difficult because many factors are known to affect the production and loss of free viruses in the plankton^3^,^23^. Nevertheless, the VPR has largely been used to infer the importance of viral-induced effects, though it has clear limitations^37^. Based on this premise, our results would imply that viruses in the clear lake are a more important source of microbial mortality than in the turbid ones. Yet, direct assessment of the importance of viral-induced mortality of microbes in glacier-turbid lakes remains challenging because glacial flour largely interferes with the observation of host infectivity by transmission electron microscopy, as well as with the use of other approaches, such as the reduction and reoccurrence of viruses. Despite these limitations, our study has described for the first time the abundance and spatiotemporal distribution patterns of VLP in turbid glacier-fed lakes and has opened several question regarding their ecological importance that need to be addressed in future research.

## Methods

### Study site

**Figure 1:**
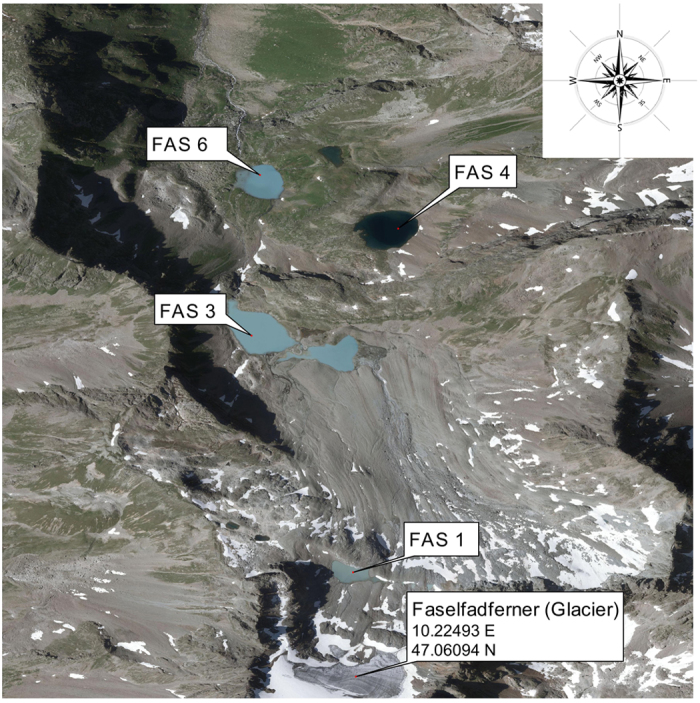
Faselfad catchment showing the three turbid lakes FAS 1 (2600 m a.s.l.), FAS 3 (2400 m a.s.l), and FAS 6 (2200 m a.s.l.), as well as the clear lake FAS4 (2400 m a.s.l.). FAS 1 and FAS 3 are connected with a stream, however, depending on time of the year this stream flows partially below surface. FAS 4 and FAS 6 are also connected by a partially subsurface stream. Orthophotograph source: http://www.tirol.gv.at/tiris.

**Figure 2:**
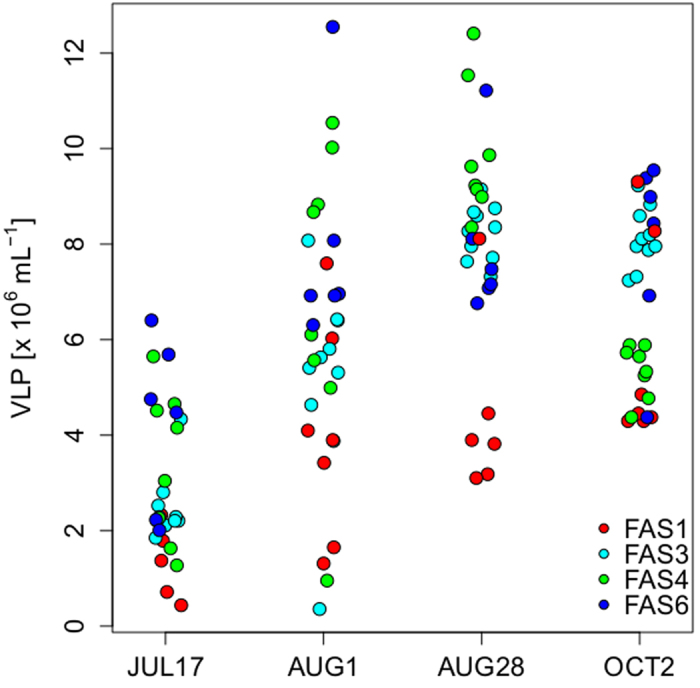
Changes in virus-like particle (VLP) abundance throughout the ice-free season. Shown are all measured samples (i.e., different depths) at four times of the year in the four Faselfad lakes. Values on x-axis are jittered for clarity.

**Figure 3:**
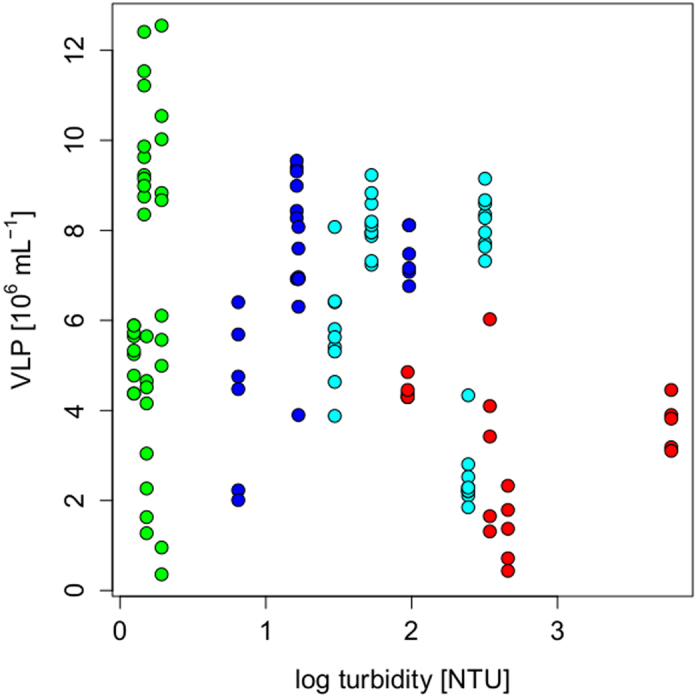
Virus-like particle (VLP) abundance across the turbidity gradient including all measured samples. Colors codes are the same as in Fig. 2.

**Figure 4:**
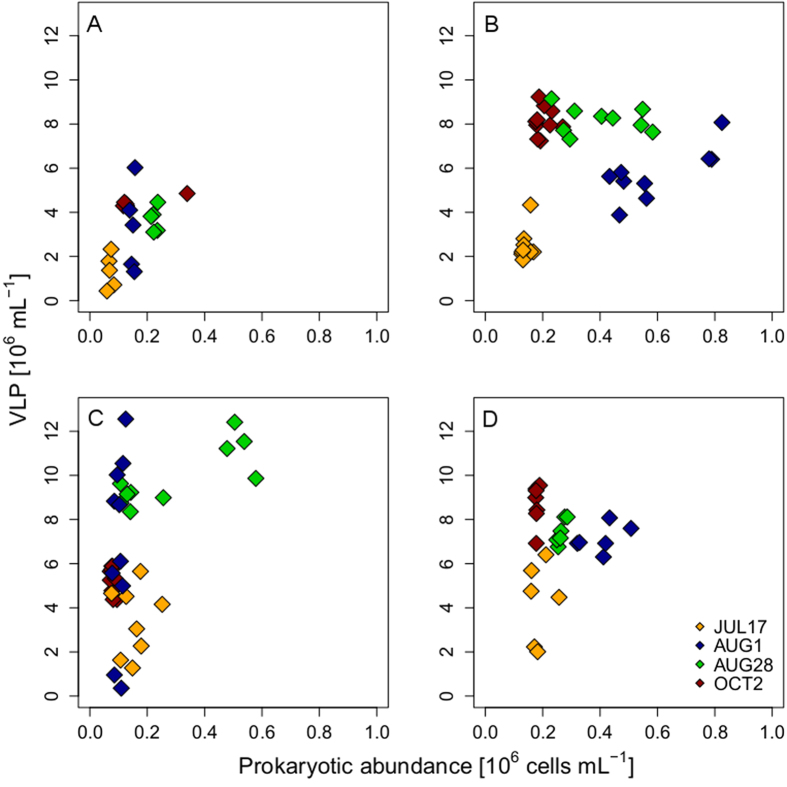
Spatiotemporal coupling between virus-like particle (VLP) and prokaryotic abundance in the four Faselfad lakes. Panel (**A**) FAS 1, (**B**) FAS 3, (**C**) FAS 4, and (**D**) FAS 6. Note the decrease in VLP abundance towards the end of the ice-free season in FAS 4 (**C**), whereas VLP abundance remained at similar levels in the turbid lakes.

**Figure 5:**
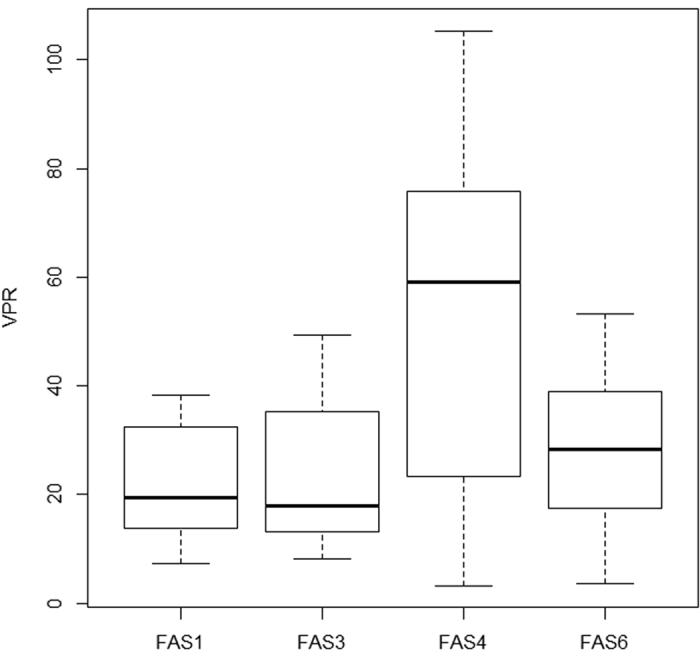
Virus-to-prokaryote ratio (VPR) including all data of the clear and turbid Faselfad lakes. The line indicates the median, the box the 25% and 75% quartiles, while the whiskers extend to the minimum and maximum values of VPR.

The study area comprises the Faselfad lakes (FAS), a group of six adjacent lakes situated between 2263 and 2620 m a.s.l. in the western Austrian Alps (47°4’ N, 10°13’E, Fig. 1). All six lakes originate from a retreating glacier, the ‘Faselfadferner’, which is rapidly receding as indicated by the comparison from old and present cartographic maps (not shown). This small glacier is on a steep slope and thus, no cryoconites are found. Fish and cladocerans are absent in the Faselfad lakes^12^,^16^,^38^. FAS 1 (4 m deep) is the youngest lake (ca. 40–50 years) and is located directly beneath the glacier terminus (i.e., proglacial)., FAS 2, and FAS 3 (2400 m a.s.l) are located 200 m below FAS 1 and are also fed by glacial melt water. These two lakes are at certain times connected and we focused our sampling on the larger and deeper FAS 3 (16 m). These lakes are highly turbid, but FAS 1 has the highest values (reaching up to 55 NTU). The two clear lakes, FAS 4 and FAS 5 (2400 m a.s.l.) are fed only by seepage water and became clear after the glacier receded below two different rock outcrops. While FAS 4 is deep (15 m), FAS 5 is shallow and potentially freezes completely during winter. Therefore, we selected FAS 4 for the sampling. In FAS 6 (2260 m a.s.l), water from lakes FAS 3, FAS 4, and 5 is pooled resulting in a reduced turbidity compared to FAS 3. The hydrological connectivity among FAS lakes 1, 3, and 6 is probably relevant for the viral patterns observed. Samples were taken during the ice-free season at four time points. Because sampling of such remote systems is weather dependent, the time gaps between samplings were different. Samples were taken on July 17, August 1, August 28, and October 2, 2012.

### Sampling

Water samples for enumeration of VLP were taken above the deepest point of each lake from a boat, with a 2 L Schindler-Patalas sampler at discrete depths. In FAS 3, 4, and 6, samples were collected at the surface, 1 m, 2 m, and then at 2 m depth intervals until the maximum depth. In the shallow lake FAS 1, samples were collected every meter.

### VLP counting efficiency

Viral abundance was enumerated using SYBR Gold staining (Invitrogen, USA) and epifluorescence microscopy following the protocol of Suttle and Fuhrman^39^. To assess the potential interference of glacial mineral particles with the counting efficiency of VLP, a preliminary experiment using solutions of different turbidity and the addition of T4 bacteriophages was conducted in triplicates. T4 phages from a stock solution (10^7 ^VLP mL^−1^) were added to a T4 buffer solution (10 mM Tris-HCl pH: 7.2; 10 mM MgSO_4_) and then dry glacial flour (0.0285 g mL^−1^ for NTU 7 and 0.0570 g mL^−1^ for NTU 14) was added and mixed. The control had only T4 phages suspended in the buffer.

### VLP abundance

Freshly collected water samples were immediately transferred into 4 mL cryovials, fixed for 15–30 min in 0.5% EM-grade glutaraldehyde (Sigma, USA) and subsequently flash frozen in liquid nitrogen. For long-term storage, samples were transferred to −80 °C until preparation. For this purpose, samples were thawed in a 37 °C water bath and then homogenized with a vortex mixer, before 1 mL was transferred into 1.5 mL Eppendorf vials (for highly turbid samples the volume was reduced to 250 μL and raised up to 1 mL with MQ water previously filtered through a 0.02 μm pore size filter). The filtration unit was mounted with a pre-wetted 0.45 μm nitrocellulose support filter and a 0.02 μm Anodisc filter (Whatman, UK) was carefully placed on top of it. The filtration funnel was clamped onto the soaked filters, the sample was vortexed for 3–5 s, immediately before dripping it slowly in circles onto the Anodisc to guarantee an even sample distribution. Using a hand-operated vacuum pump (≤13 kPa), the samples were slowly filtered onto the Anodisc filter, which was then carefully removed while vacuum was still on and after it became dry, it was stained with SYBR Gold. Stained virus-like particles (VLP) were counted with an epifluorescence microscope (Axiophot 2, Zeiss, Germany) at 1250–2500x magnification.

### Estimating the effect of free DNA on VLP counting

Free DNA in the water column might be confused with small VLPs and lead to overestimates of VLP abundance. Following the protocol of Bettarel *et al.*^40^, samples from each lake were treated with DNase I (Sigma-Aldrich, USA) dissolved in 1 mL 0.15 M NaCl. Treatments consisting of 875 μL lake water and 125 μL DNase were incubated with 250 Kunitz units of DNase for 30 min at 25 °C. A negative control consisting of 875 μL lake water and 125 μL MQ water was included. Samples were then stained and counted following the SYBR Gold protocol described above.

### Induction of prophages

To assess the importance of the lysogenic life cycle, we conducted two experiments where prophages were chemically induced following the protocol recommended in Paul & Weinbauer^41^. On August 1 and October 2, composite samples (i.e., the same volume pooled from the different depths) from FAS 3 and FAS 4 were collected and mitomycin C (Sigma) was added to 25 mL of lake water (final concentration: 0.5 μg mL^−1^). The samples were incubated for 24 h at room temperature and then fixed with 2% glutaraldehyde. Controls were fixed with formaldehyde (final concentration 2%) prior (3 h) to the addition of mitomycin C. The VLP abundance in the control and treatments were counted as described above.

### Auxiliary parameters

Turbidity, total dissolved phosphorus (TDP) and chlorophyll-a were measured in three independent composite samples collected in parallel at each lake as described in Peter & Sommaruga^12^, whereas dissolved organic carbon (DOC), dissolved nitrogen (DN), prokaryotic abundance, and water temperature were measured at discrete depths as described for the viral samples. Turbidity was measured as nephelometric turbidity units (NTU) using a Turb 430 T (WTW, Germany), which was calibrated between 0.02, 10, and 1000 NTU. This instrument is tailored to measure the turbidity given by small particles such as those glacial ones. For each sample, three individual measurements were conducted and the average was calculated. TDP concentrations were measured in filtered samples (Whatman GF/F filters) by spectrophotometry following the molybdate method after digestion with sulfuric acid and hydrogen peroxide. Chlorophyll-a extraction from 3 L water samples filtered on Whatman glass fiber GF/F filters was done with acetone and the equation of Lorenzen^42^ was used to calculate its concentration. The DOC concentration (as non purgeable organic carbon) was measured in 40 mL subsamples with a Total Organic Carbon Analyzer (TOC-Vc series, Shimadzu, Japan) using a calibration curve from 0.1 to 1 mg L^−1^. The samples were previously filtered through two glass-fiber filters (Whatman, GF/F; precombusted for 2 h at 450 °C) using a stainless-steel syringe holder. The filters and the syringe holder were rinsed with 20 mL of MQ and 10 mL of sample water. Filtered samples were collected in precombusted (4 h at 450 °C) glass vials (40 mL, Shimadzu), acidified with HCl to pH 2, and stored in the dark at 4 °C until analysis (within 48 h). Three to five injections were analyzed for each sample. In the same run, DN was measured in a total nitrogen measuring unit (Shimadzu TNM–1).

We used flow cytometry and nucleic acid staining with SYTO13 to enumerate prokaryotes^43^ in formaldehyde-fixed samples (2% final conc.). For this, 1 mL of sample was stained with SYTO-13 (final concentration: 2.5 μM) for 10 min at room temperature in the dark. Fifty μL of fluorescent beads (1 μm diameter Sigma Aldrich), which were previously enumerated under the epifluorescence microscope, were added to each sample as a reference. Samples were analyzed on a flow cytometer (MoFlo Astrios, Beckman Coulter, USA) using forward scatter as trigger and log-transformed fluorescence and side scatter signals of a 488 nm laser to target prokaryotes. Cell abundance was calculated as the ratio of beads to prokaryotes and compensated for dilution with the fixative and dye.

Water temperature was measured with a thermometer (±0.1 °C) placed inside the water sampler.

### Statistics

Most statistical analyses were calculated with PAST^44^, such as ANOVA (with Tukey’s pairwise posthoc comparison), Welch F test in case of violation of the equal variance assumption of ANOVA, and multiple linear regression analysis. Figures were prepared using R^45^.

## Additional Information

**How to cite this article**: Drewes, F. *et al.* Are viruses important in the plankton of highly turbid glacier-fed lakes? *Sci. Rep.* **6**, 24608; doi: 10.1038/srep24608 (2016).

## Supplementary Material

Supplementary Information
