# Corrigendum: Are viruses important in the plankton of highly turbid glacier-fed lakes?

**DOI:** 10.1038/srep28407

**Published:** 2016-07-07

**Authors:** Fabian Drewes, Hannes Peter, Ruben Sommaruga

Scientific Reports 6: Article number: 2460810.1038/srep24608; published online: 04202016; updated: 07072016

In this Article, references 30 and 31 are not listed in the correct order. The correct references are listed below:

30. Knowles, B. *et al*. Lytic to temperate switching of viral communities. *Nature* **531**, 466–470, doi: 10.1038/nature17193 (2016).

31. Jacquet, S. & Bratbak, G. Effects of ultraviolet radiation on marine virus-phytoplankton interactions. *FEMS Microbiol. Ecol.* **44**, 279–289, doi: 10.1016/s0168-6496(03)00075-8 (2003).

