# Supplementary material for: Are viruses important in the plankton of highly turbid glacier-fed lakes?
Source: Sci Rep. 2016 Apr 20;6:24608. doi: 10.1038/srep24608 (PMC4837353; doi:10.1038/srep24608)
Supplement: Supplementary Information [file srep24608-s1.pdf]

Supplementary Material

Are viruses important in the plankton of highly turbid glacier-fed lakes?

Fabian Drewes, Hannes Peter, and Ruben Sommaruga

University of Innsbruck, Institute of Ecology, Lake and Glacier Research Group, Technikerstr. 25, 6020 Innsbruck, Austria

Content:

Page 1: Supplementary Material, Figure S1. Abundance of the T4 bacteriophage in the experiment to test counting efficiency.

Page 2: Supplementary Material, Figure S2. Relative loss in VLP counts after treatment with DNase.

Page 3: Supplementary Material, Figure S3. Variability in VLP abundance in the water column of the four Faselfad (FAS) lakes and in relation with water temperature.

Page 4: Supplementary Material, Figure S4. Depth distribution of VLPs during the ice-free season in the four Faselfad (FAS) lakes.

Page 5: Supplementary Material, Figure S5. Relative changes in VLP abundance following prophage induction with mitomycin C for 24 h.

Page 6: Supplementary Table S1. Key environmental parameters of the Faselfad lakes during the ice-free season 2012.

Page 7: Supplementary Table S2. Summary of the multiple linear regression model.

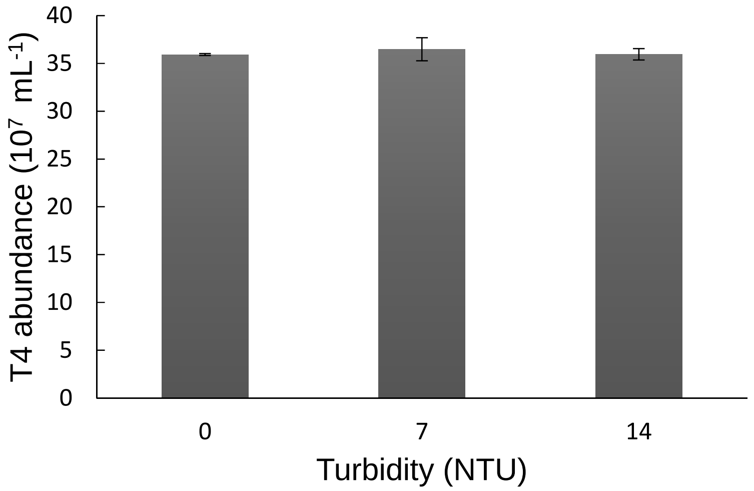

Supplementary Material, Figure S1. Abundance of the T4 bacteriophage in the experiment to test counting efficiency. Phage enumeration in the control (0 nephelometric turbidity unit, NTU) and in the treatments (7 and 14 NTU) was done by epifluorescence microscopy after staining with SYBR Gold. Error bars indicate ±1SD. n=3.

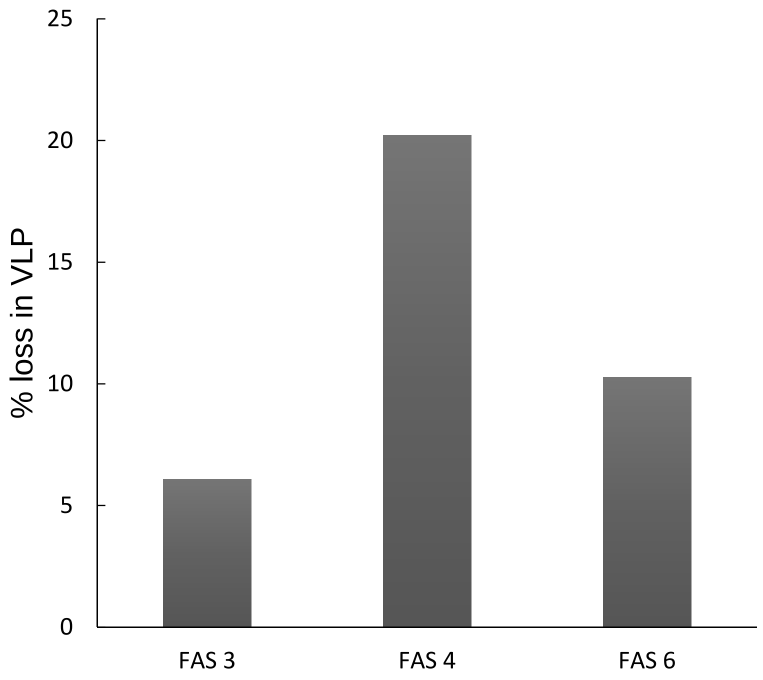

Supplementary Material, Figure S2. Relative loss in VLP counts after treatment with DNase. Results are expressed as percentage, considering 100% the respective control in each Faselfad lake.

A

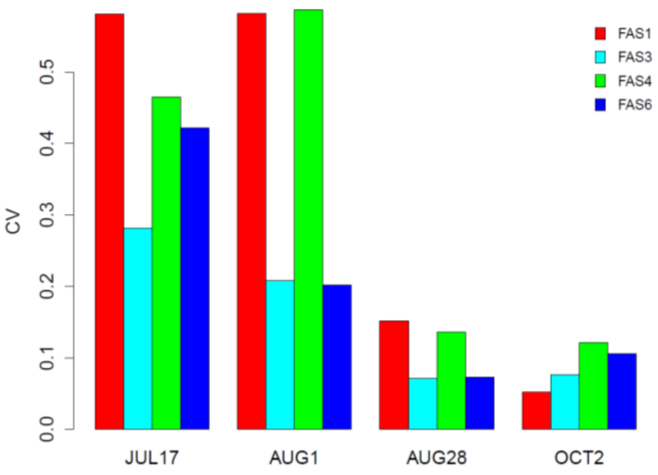

B

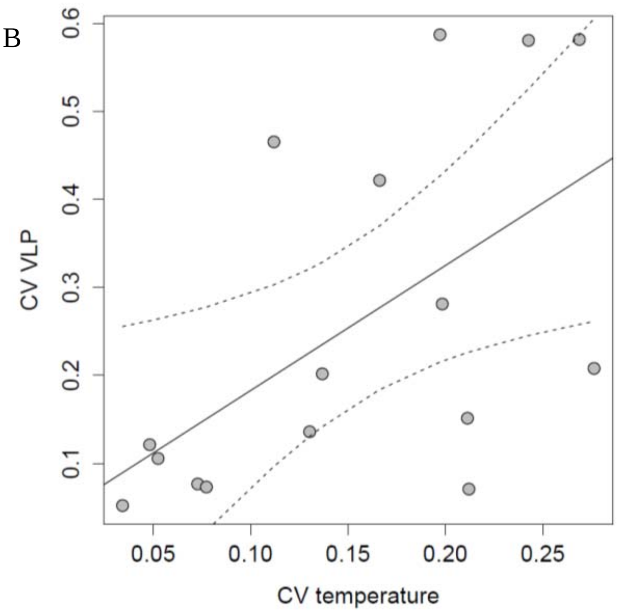

Supplementary Material, Figure S3. Variability in VLP abundance in the water column of the four Faselfad (FAS) lakes and in relation with water temperature. Panel A: coefficient of variation in VLP counts for each lake and sampling date as an indicator for the distribution homogeneity of VLPs across the water column. Small values indicate an even distribution, whereas larger values indicate larger changes in VLP distribution across the water column. **Panel B:** Variation of VLP was positively related to variation in lake water temperature.

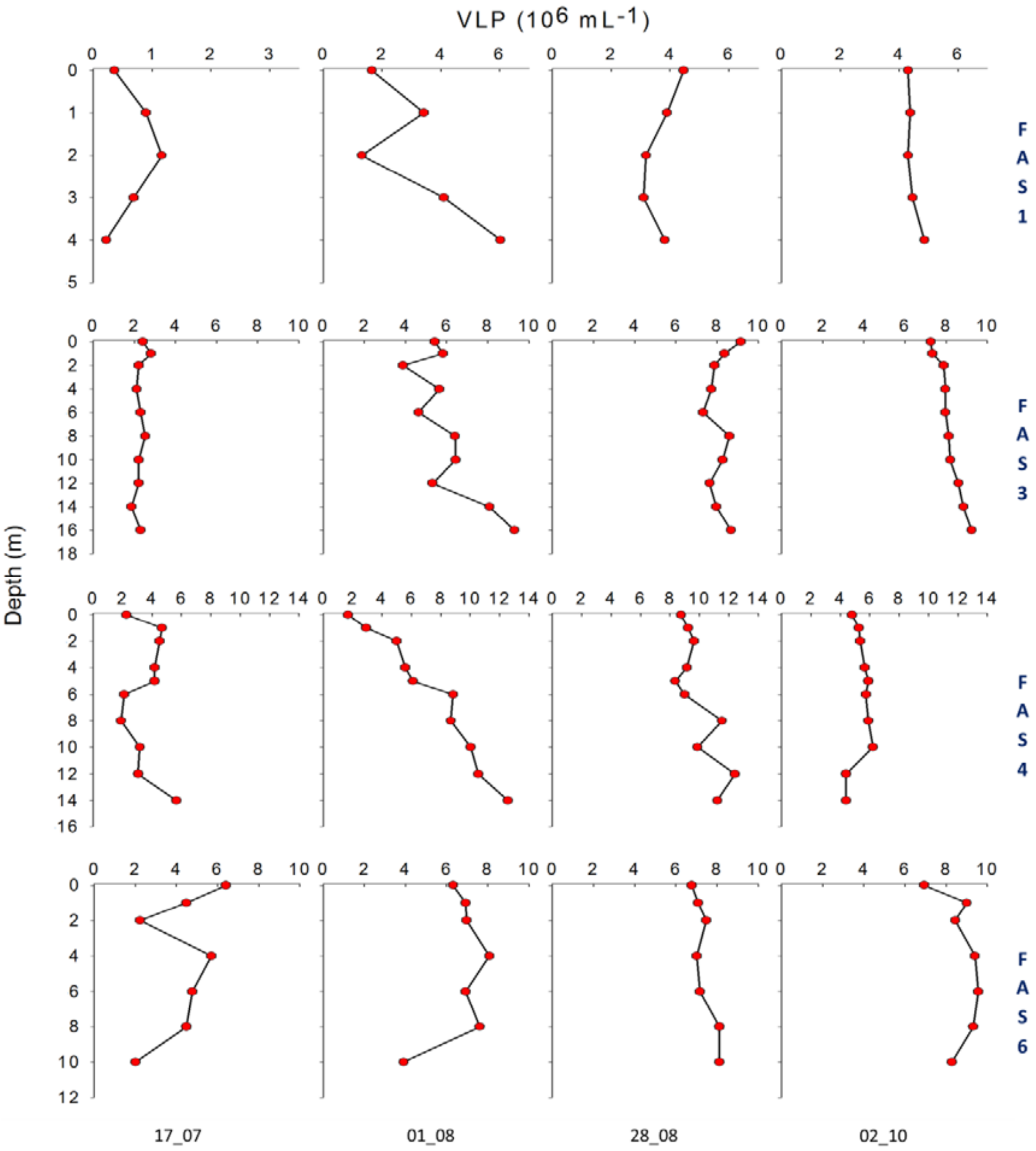

Supplementary Material, Figure S4. Depth distribution of VLPs during the ice-free season in the four Faselfad (FAS) lakes.

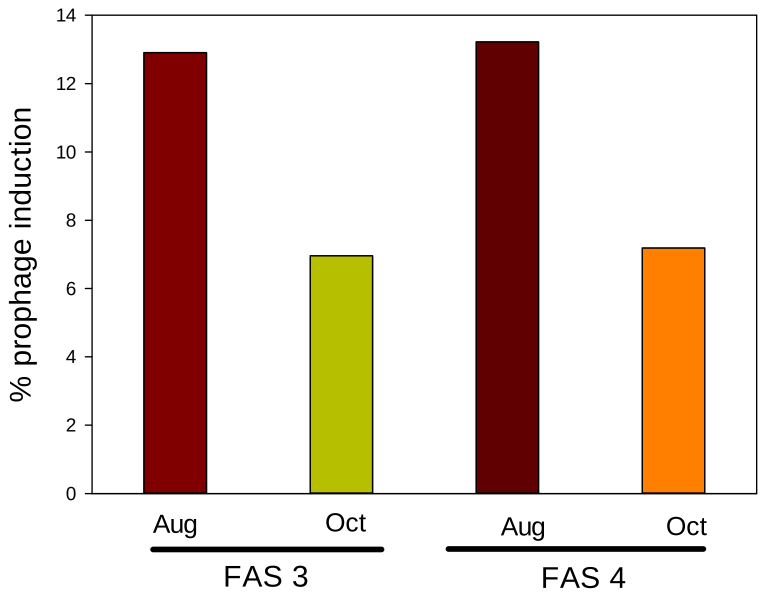

Supplementary Material, Figure S5. Relative changes in VLP abundance following prophage induction with mitomycin C for 24 h. Experiments were done with composite water samples collected from FAS 3 and FAS 4 on August 1 and October 2. A killed control was included on each experiment before adding mitomycin C.

Supplementary Table S1. Table 1. Key environmental parameters of the Faselfad lakes during the ice-free season 2012. Given are mean ± standard deviation values over the water column (Water temperature, DOC, DN and Prokaryotic abundance, n_{FAS1} = 5, n_{FAS3} = 10, n_{FAS4} = 10, n_{FAS6} = 7) or mean ± standard deviation measured from composite water samples (Turbidity, TDP, Chlorophyll-a, n=3).

Site	Date	Water temperature [° C]	Turbidity [NTU]	DOC [µg L^{-1}]	TDP [µg L^{-1}]	DN [µg L^{-1}]	Chlorophyll-a [µg L^{-1}]	Prokaryotic abundance [10^5 cells mL^{-1}]
FAS 1	07/17	2.09±0.48	13.3±0.07	181.6±7.6	2.3±0.17	161.0±0.8	0.12±0.0	0.68±0.11
	08/01	4.04±1.09	11.6±0.74	215.4±97.3	3.5±0.3	154.6±8.3	0.13±0.05	1.48±0.08
	08/28	4.64±0.98	42.8±5.67	284.2±90.8	4.6±0.15	185.3±9.4	0.13±0.07	2.27±0.10
	10/02	3.93±0.13	6.2±4.36	182.8±106.7	2.5±0.21	266.0±15.4	NA	1.59±0.88
FAS 3	07/17	5.59±1.11	9.9±1.30	185.9±50.4	1.5±0.1*	161.8±1.7	0.99±0.16	1.46±0.19
	08/01	6.86±1.90	3.4±0.36	200.4±44.5	1.5±0.17	141.2±5.2	1.14±0.05	6.10±1.39
	08/28	7.63±1.62	11.2±7.89	323.8±69.1	2.5±0.3	148.4±7.0	4.57±0.27	3.89±1.25
	10/02	5.55±0.40	4.6±0.48	259.5±66.6	1.5±0.69	183.2±9.9	NA	2.05±0.30
FAS 4	07/17	6.29±0.70	0.2±0.04	219.3±32.4	0.7±0.0	191.3±2.0	0.37±0.06	1.52±0.46
	08/01	7.92±1.56	0.3±0.03	249.4±32.1	0.9±0.21	175.5±13.1	1.15±0.11	1.02±0.15
	08/28	9.72±1.27	0.2±0.01	440.3±118.9	2.0±1.05	181.5±10.9	1.21±0.12	3.03±1.91
	10/02	6.76±0.33	0.1±0.0	316.8±23.8	0.6±0.12	187.1±9.6	NA	0.79±0.07
FAS 6	07/17	6.53±1.09	1.3±0.22	244.8±38.2	1.0±0.0	138.7±1.7	0.47±0.18	1.86±0.36
	08/01	8.30±1.14	2.4±0.20	221.9±29.0	1.5±0.64	149.2±14.8	0.45±0.13	4.10±0.66
	08/28	9.02±0.70	6.3±0.64	363.5±34.6	1.3±0.23	150.7±8.2	4.36±0.17	2.70±0.18
	10/02	5.86±0.31	2.4±0.25	303.4±56.9	1.1±0.21	169.5±10.8	NA	1.77±0.08

* 1 outlier removed (n=2)

Supplementary Table S2. Summary of the multiple linear regression model to estimate the importance of recorded factors on VLP abundance. All measured values of turbidity, prokaryotic abundance, water temperature, and DOC were included in the model, which yielded an overall adjusted multiple determination coefficient (R^2 adjusted) of 0.42 and was significant (ANOVA, $F = 22.96$, $p < 0.01$).

	Coeff.	Std.err.	t	p	R^2
Constant	-30034	7.79E+05	-0.04	0.97	0
Turbidity	-28717	18785	-1.53	0.13	0.03
Prokaryotic abundance	0.57	0.11	5.02	< 0.01**	0.15
Temperature	3.21E+05	1.12E+05	2.88	< 0.01**	0.25
DOC	10026	2262.9	4.43	< 0.01**	0.22

** Significant factors in the multiple linear regression model
